# Draft Genome Sequence of a Phytopathogenic Ganoderma sp. Strain That Causes Basal Stem Rot Disease on Oil Palm in Sabah, Malaysia

**DOI:** 10.1128/MRA.01240-19

**Published:** 2020-01-02

**Authors:** Christopher L. Y. Voo, Daniel E. T. Yeo, Khim-Phin Chong, Kenneth F. Rodrigues

**Affiliations:** a Biotechnology Research Institute, Universiti Malaysia Sabah, Kota Kinabalu, Sabah, Malaysia; b Faculty of Science and Natural Resource, Universiti Malaysia Sabah, Kota Kinabalu, Sabah, Malaysia; University of California, Riverside

## Abstract

Basal stem rot (BSR) disease on Elaeis guineens is known to be caused by members of the pathogenic fungal genus *Ganoderma*, especially the species Ganoderma boninense. This species affects oil palm plantation in Sabah, Malaysia. The genome sequence (52.28 Mbp) will add to the representation of this genus, especially in regard to BSR disease.

## ANNOUNCEMENT

Members of the *Ganoderma* genus are most famous in Asia for their medicinal properties and for causing basal stem rot (BSR) disease. A major economic concern in the state of Sabah, Malaysia, would be the effect of BSR disease on the palm oil industry. As of 2015, 27% of the areas used for oil palm plantation in Malaysia are in Sabah (www.nepcon.org). The estimated economic loss due to the disease in Malaysia for a period of 6 months could be up to 43.32% (1). Given the availability of multiple *Ganoderma* sp. genomes (2–4), we are hopeful that a genetic solution to the disease that has broad effectiveness across multiple species of the genus can be found.

The fungal specimen (BRIUMSc) was obtained from infected oil palm trees in the north of Sabah in 2010. The fungal mycelia were aseptically transferred to multiple potato dextrose agar (PDA) plates. The mycelia were grown for 2 to 3 weeks from a fixed middle point on the PDA plate. This ensures that mycelial growth could be tracked from that one point with a consistent colony morphology, and any plates with nonpredetermined colony growth or obvious fungal contamination were discarded.

Genomic DNA was extracted using the cetyltrimethylammonium bromide (CTAB) extraction protocol (5). The extracted DNA was first used in the molecular identification of the species based on the internal transcribed spacer 1 (ITS1)-ITS2 region of the rRNA genes; the sequences were deposited in GenBank under the accession no. JN234429. The sequence percent similarity (NCBI BLASTN 2.9.0, June 2019) to Ganoderma boninense ranges from 99.67% to 98.27%; however, within this range are also species identified as Ganoderma orbiforme and Ganoderma miniatocinctum. This was followed by whole-genome sequencing (WGS) using the Illumina HiSeq platform. A target library size of 500 bp was selected, and the genomic sequencing coverage aim was to achieve close to 100×. The genome was fragmented based on the sonication shear method, and paired-end sequencing to 100 bp was applied. Greater than 5 Gbp of sequenced data were targeted based on an estimated genome size of 50 Mb from similar genomes of Basidiomycota available at the time. The resulting data were quality filtered with a minimal 100-bp length at Q30 and above before proceeding to *de novo* assembly using CLC Genomic Workbench version 12.0. The parameters set in the program were the following: word size, 45; bubble size, 98; and perform scaffolding, default settings for “map reads back to contigs,” with “global alignment” option, and with “update contigs” option.

The assembled draft genome is 52.28 Mb, with a G+C content of 55.63%, 99.35× sequencing coverage, 12,153 contigs/scaffolds, an *N*_50_ length of 6,787 bp, and a maximum scaffold size of 132,168 bp. It was generated from approximately 52 Gbp of sequenced data, corresponding to 52 million (95.29% of the total) sequenced reads, of which 45 million are paired. Although this draft genome sequence is rather fragmented, BUSCO version 3 analysis (6) with the associated AUGUSTUS program (7) indicates that 90.26% (1,205 of 1,335 genes) of the orthologous genes from the ortholog database (Basidomycota_odb9) are represented. This consists of 994 genes with complete representation and 211 genes with fragmented representation, while another 130 genes are missing. The BUSCO annotation results are 74.5% (single-copy genes [S], 70.9%; duplicated genes [D], 3.5%); fragmented genes (F), 15.8%; missing genes (M), 9.7%; and number of genes used (n), 1,335.

### Data availability.

This whole-genome shotgun project has been deposited in GenBank under the accession no. VJXU00000000, SRA accession no. SRX6934198, and BioProject accession no. PRJNA553124. The version described in this paper is the first version, VJXU01000000.
